# Acute effects of exergaming on young adults’ energy expenditure, enjoyment, and self-efficacy

**DOI:** 10.3389/fpsyg.2023.1238057

**Published:** 2023-08-14

**Authors:** Dandong Gu, Chenling Gu, John Oginni, Suryeon Ryu, Wenxi Liu, Xianxiong Li, Zan Gao

**Affiliations:** ^1^School of Physical Education, Hengyang Normal University, Hengyang, China; ^2^Central South University of Forestry and Technology, Changsha, China; ^3^Department of Kinesiology, Recreation, and Sports Studies, University of Tennessee, Knoxville, TN, United States; ^4^School of Kinesiology, University of Minnesota–Twin Cities, Minneapolis, MN, United States; ^5^Department of Physical Education, Shanghai Jiao Tong University, Shanghai, China; ^6^School of Physical Education, Hunan Normal University, Changsha, China

**Keywords:** accelerometers, college students, Just Dance, exergaming, perceived enjoyment, physical activity, self-efficacy

## Abstract

This study investigated the effects of a dance-based exergaming on Chinese college students’ energy expenditure, self-efficacy, and enjoyment in comparison with the traditional aerobic dance exercise. Forty young adults (33 females; Mage = 21.55 years, SD = 2.06) completed two separate 20 min exercise sessions with 10 min intervals on the same day: (1) Xbox 360 Kinect Just Dance exergaming session; and (2) a traditional instructor-led aerobic dance exercise session. Participants’ energy expenditure (Kcal/session) was measured by the ActiGraph GT9X Link accelerometers, and their perceived self-efficacy and enjoyment were assessed via validated surveys following each session. Dependent t-test indicated significant differences in participants’ enjoyment (*t* = −1.83, *p* = 0.04). Specifically, participants in the dance-based exergaming session reported a higher level of enjoyment (M = 3.96, SD = 0.65) as compared to the aerobic dance session (M = 3.61, SD = 0.54). However, there was no significant difference in energy expenditure and self-efficacy between the two sessions. Findings suggest that college students had comparable energy expenditure as the traditional aerobic dance session while experiencing more fun and enjoyment. This suggests that exergaming can be a fun exercise alternative for promoting physical activity among young adults.

## 1. Introduction

Physical inactivity and sedentary behavior can have a severe negative impact on adults. It can increase the risk for heart disease, stroke, high blood pressure, Type 2 diabetes, and certain types of cancer (Santos-Lozano et al., 2021; Sheng et al., 2021; Xiao and Rosenzweig, 2021; Zhu, 2022). It can also lead to mental health issues such as depression, anxiety, and low self-esteem (Gao and Lee, 2022). Furthermore, physical inactivity and sedentary behavior can also lead to weight gain, joint pain, poor balance and coordination, and weak muscles (Pellegrini et al., 2016; Lee et al., 2019). Therefore, it is essential to make physical activity and a healthy lifestyle a priority to maintain overall health and well-being in young adults, including college students. As known, regular physical activity helps to maintain a healthy weight and lowers blood pressure, which reduces the risk of developing type 2 diabetes, heart disease, and other chronic diseases (Jennings et al., 1989; Chen et al., 2022; Gao and Lee, 2022). By staying active, college students can improve their overall physical fitness and reduce their risk of developing chronic diseases (Vainshelboim et al., 2019). Regular physical activity can also help reduce stress, improve mood, and increase energy levels (Jennings et al., 1989; Chen et al., 2022; Gao and Lee, 2022). College students can engage in physical activities such as running, jogging, and cycling to help them stay fit and healthy.

Physical activity engagement among college students has been on a downward trend in recent years. Studies have found that only a small percentage of college students are meeting the recommended amount of physical activity and that most are not engaging in any physical activity at all (Douglas et al., 1997; Ferreira Silva et al., 2022). For example, recent studies have shown that physical activity engagement in Chinese college students is declining (Yuan et al., 2018; Wang, 2019; Bao et al., 2020). With the growing prevalence of digital devices and the development of technology, physical activity has been increasingly replaced by sedentary activities. This has resulted in the decrease of physical activities among Chinese college students (Yuan et al., 2018; Xu et al., 2021). Additionally, due to the high academic pressure and the lack of leisure time, students are not motivated to exercise, leading to an overall decrease in physical activity (Li et al., 2022). Although there are some efforts to encourage physical activity, such as the launch of various sports activities and the construction of sports facilities on campuses, the current physical activity engagement level among college students in China is still too low. As a result, college campuses should now make an effort to create more accessible and engaging opportunities for physical activity, such as intramural sports, outdoor recreation, and exergaming activities and events.

In the past decade, exergaming has proven to be an effective way to promote physical activity and health in individuals (Douris et al., 2012; Gao et al., 2012; Gao and Xiang, 2014; Benzing and Schmidt, 2018; Ye et al., 2019; Gonçalves et al., 2021; Baysden et al., 2022; Pasco and Roure, 2022; Sadeghi and Jehu, 2022; Sousa et al., 2022). Exergaming combines video gaming with physical activity, which helps to make exercise more enjoyable and accessible. It provides an alternative to traditional physical activity, making it more appealing and accessible to college students (Siegel et al., 2009; McDonough et al., 2018, 2020). Research has shown that exergaming can be an effective way to encourage individuals to become more physically active in college students (McDonough et al., 2018). Research has found that exergaming can increase energy expenditure among players (Gao et al., 2019; O'Loughlin et al., 2020; Mohd et al., 2021). This can be especially helpful for college students who may not have the time or resources to fit in regular exercise and can benefit from a fun and engaging activity.

Studies have shown that exergaming can lead to increased motivation, self-efficacy, and enjoyment in college students (Siegel et al., 2009; Liu et al., 2019; McDonough et al., 2020; Pasco and Roure, 2022; Sousa et al., 2022). It has been found that college students who regularly engage in exergaming have higher levels of self-efficacy and an improved sense of enjoyment in physical activity (McDonough et al., 2018, 2020). Additionally, the interactive and social components of exergaming can help create a sense of camaraderie among participating students. Furthermore, exergaming can provide an engaging and fun way to meet the recommended levels of physical activity in college students. Overall, exergaming is an effective tool for promoting self-efficacy and enjoyment in college students.

Recent studies have compared outcome variables of various exergaming modalities (e.g., virtual reality, and Xbox) to traditional exercise (e.g., cycling) (Zeng et al., 2017; McDonough et al., 2018; Liu et al., 2019; McDonough et al., 2020; Zeng et al., 2022). Taking into consideration the literature reviews and the limitations noted in earlier studies, we understand that different physical activity modalities may have varying effects on individuals’ physiological and psychological outcomes. However, few studies compare the effects of exergaming aerobic dance and traditional aerobic dance among college students (Gao et al., 2013). This study aims to compare the effects of exergaming aerobic dance to traditional aerobic dance on the physiological and psychological outcomes of young adults. We hypothesize that energy expenditure will be similar, but that self-efficacy and enjoyment will be higher when using exergaming aerobic dance. The results of this study could provide health professionals, physical educators, and young adults with a greater range of activities to promote physical activity and help to identify which exergaming programs are most effective in promoting self-efficacy and enjoyment while sustaining energy expenditure.

## 2. Materials and methods

### 2.1. Participants and the research setting

In the summer of 2018, forty young adults (33 females, 7 males) were recruited from a Southcentral Chinese University. The inclusion criteria were that participants must be enrolled at the university, aged between 18 and 25 years, in good health (i.e., no physical or mental diseases/conditions preventing PA participation), and had provided their informed consent. University research ethics approval and participant written consent forms had been obtained prior to data collection.

**Figure 1:**
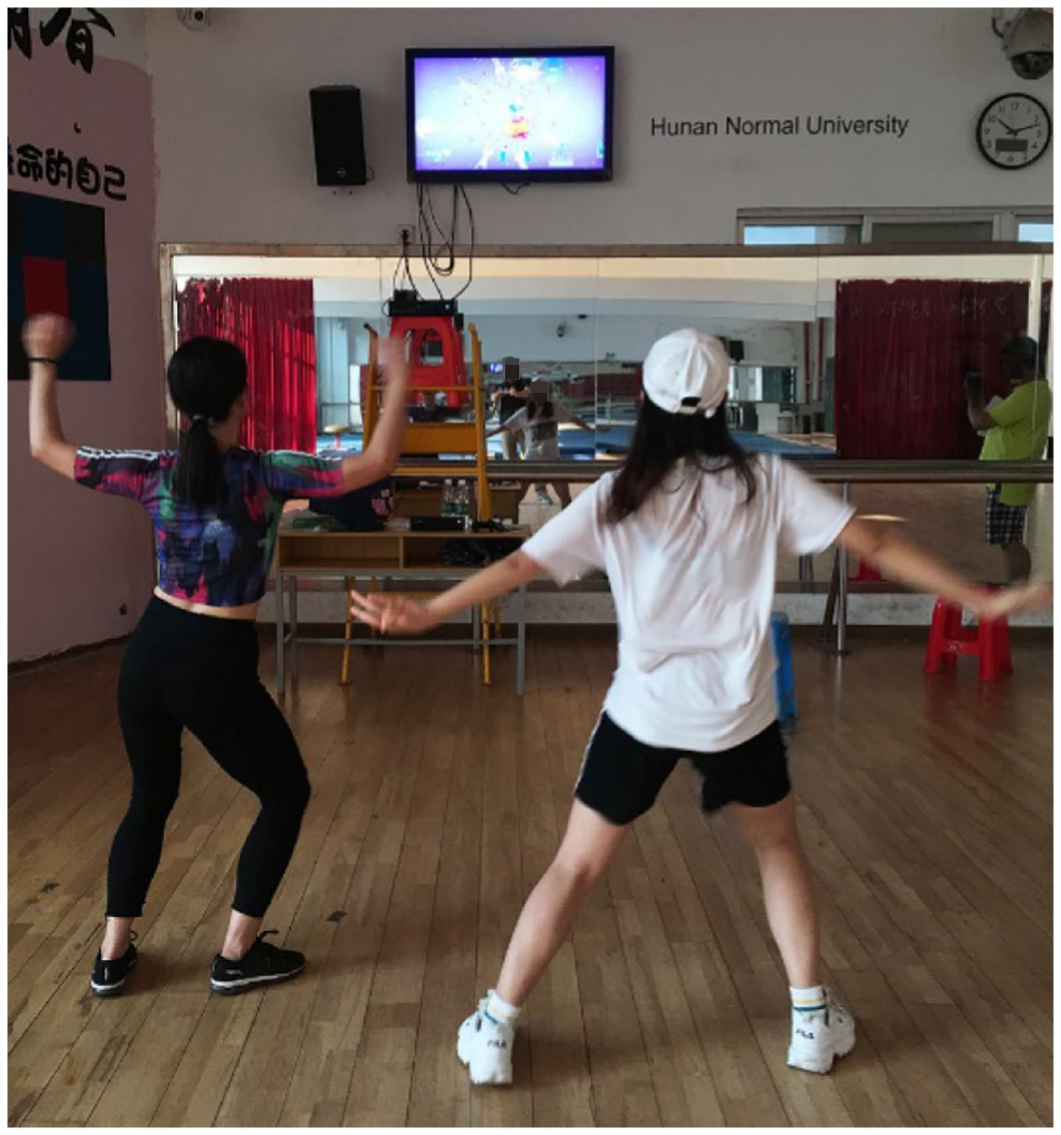
Exergaming dance.

**Figure 2:**
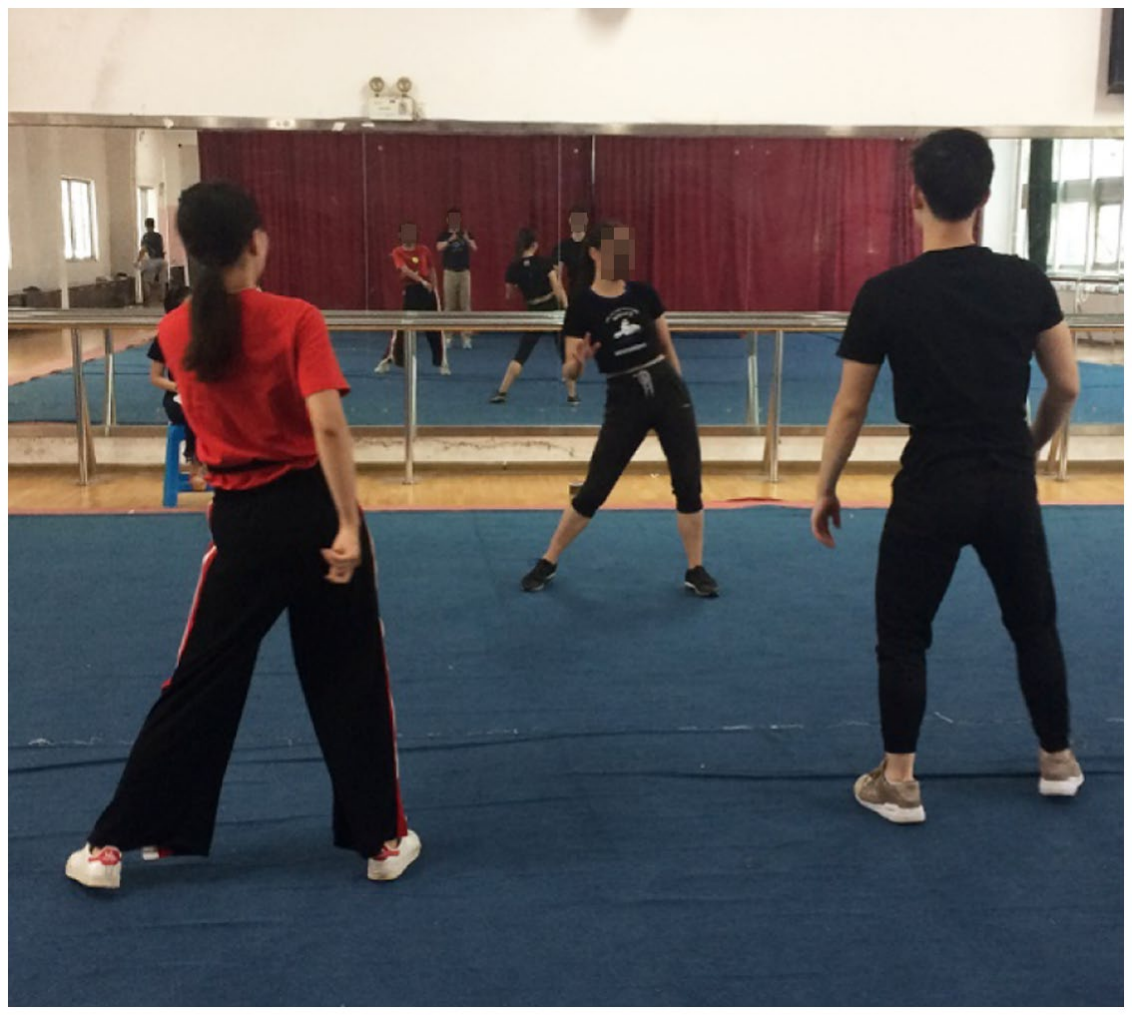
Traditional aerobic dance.

During the scheduled time slots at the campus fitness gym, those who agreed to participate completed two separate 20 min dance sessions: (1) non-stop exergaming aerobic dance (Xbox 360 Kinect Just Dance – Just Sweat around the World); and (2) traditional aerobic dance led by an experienced instructor. Specifically, Xbox 360 Kinect Just Dance is an interactive gaming experience that combines the technology of the Xbox 360 Kinect sensor with the popular rhythm-based game Just Dance. The Xbox 360 Kinect sensor allows players to control the game through their body movements, eliminating the need for traditional controllers. This innovative technology creates a more immersive and engaging gameplay experience, as players can dance, jump, and move in time with the on-screen characters. With a range of popular songs and dance routines to choose from, players can showcase their skills and compete against friends and family in a variety of game modes. Whether the player is a seasoned dancer or a complete beginner, Xbox 360 Kinect Just Dance offers a fun and accessible way to get moving and improve an individual’s coordination. In the present study, Just Dance – Just Sweat around the World was selected, as it was the perfect game for those young adults who loved to dance and wanted to incorporate a fun and challenging workout into their routines. Developed by Ubisoft, this specific game features a range of high-energy dance routines set to popular songs from around the world. With the Just Sweat mode, players can track their calories burned and set personalized fitness goals, making it a great way to stay motivated and improve overall fitness. The game can be played alone or with friends, and with a variety of difficulty levels and dance styles. In this study, participants were paired up to play the exergaming dance (See Figure 1). On the other hand, in the present study, the traditional aerobic dance session was led by an experienced instructor in the fitness gym. As known, traditional aerobic dance is a popular form of exercise that has been enjoyed by adult fitness enthusiasts for decades. With its high energy music, choreographed routines, and enthusiastic instruction, aerobic dance offers an engaging and effective way to improve cardiovascular health, burn calories, and increase muscle strength and endurance. The aerobic dance workout involved a series of choreographed dance routines that incorporate a range of movements such as jumping jacks, grapevines, and side-to-side shuffles. The instructor demonstrated the moves, provided verbal cues and encouragement, and adjusted the intensity of the workout to suit the needs of the participants. In this study, two participants were paired up to engage in aerobic dance activities led by the experienced instructor (See Figure 2).

To ensure that the results of this study were not influenced by the order in which participants were tested, we employed a counterbalancing technique. All study procedures were conducted following the ethical standards set out in the 1964 Helsinki Declaration and its later amendments (World Medical Association, 2013) and were approved by the Institutional Review Board at the University. Written informed consent was obtained from each participant before data collection.

### 2.2. Procedures

Before beginning the dance sessions, trained research assistants collected participants’ demographic and anthropometric information. Participants were randomized split into two groups and completed a 20 min exergaming dance session and a 20 min instructor-led aerobic dance sessions (See Figures 1, 2) in a counterbalanced order. To avoid potential carry-over effects between sessions, participants had a 10 min break to allow their blood pressure and heart rate to return to baseline levels. They also wore accelerometers on their hips before the exercise sessions and responded to a questionnaire evaluating their perceived self-efficacy and enjoyment following each dance session during the breaks.

The exergaming session took place at a console station set up on one side of the fitness gym and utilized Xbox 360 Kinect Just Dance — Just Sweat Around the World, including 4–5 moderate intensity dance exercise in a row. Participants followed the actual dance moves of the dancer that appeared in the game, with no transition time between dances as set by the game console. The aerobic dance session was conducted on the other side of the fitness lab, with a well-trained instructor guiding participants through a series of aerobic dances. The instructor led the dance exercise at the moderate intensity which was the same or similar level of Just Dance.

### 2.3. Measures

#### 2.3.1. Demographic and anthropometric data

On a demographic questionnaire, participants provided their date of birth, sex, college classification, and race/ethnicity. Height was determined to the closest 0.1 cm with the aid of a Seca stadiometer (Seca, Hamburg, Germany), while weight and body fat percentage were measured using the InBody 230 Body Composition Analyzer (Biospace, Seoul, Korea). Subsequently, BMI was computed as weight [kg]/height [m2] (Centers for Disease Control and Prevention, n.d.). Participants were also asked to report their prior experience with playing exergaming.

#### 2.3.2. Energy expenditure

In this research, the participants wore an ActiGraph Link GT9X accelerometer (ActiGraph, Pensacola, FL, United States) on the right hip to measure energy expenditure. The ActiGraph Link GT9X accelerometer is a small wearable device used to measure physical activity and movement patterns. It is commonly used in research studies to assess physical activity levels and sedentary behavior in pediatric and adult populations. The device is compact and light-weight, weighing only 16 grams, and can be worn on the wrist, hip, or ankle. It uses triaxial accelerometry, which measures movement in three dimensions, to capture detailed information about physical activity patterns, including intensity, frequency, and duration. The device is equipped with a range of features, including a customizable sampling rate, activity classification algorithms, and the ability to measure sedentary behavior, sleep quality, and energy expenditure. It also includes a built-in triaxial gyroscope and magnetometer, allowing for more precise movement tracking. Overall, the ActiGraph Link GT9X accelerometer is a valid and reliable tool for measuring physical activity and energy expenditure, making it a valuable tool for researchers, clinicians, and healthcare professionals in a wide range of settings (McDonough et al., 2018; Zeng et al., 2022). In the present study, activity intensity can be classified into different categories based on established cut points, as the sum of activity counts in an epoch shows a linear relationship with activity intensity.

Given the short duration of the exercise session and the aims of this study, activity counts were measured in 1-s epoch, and physical activity levels were quantified as average activity counts per 1 s for activity intensities. Cut points established by Freedson adult VM3 (1998) were applied to the data. In detail, for the ActiGraph accelerometer, the cut points are 100 counts per minute (cpm) for sedentary behavior, 100–1951 cpm for light activity, 1952–5,724 cpm for moderate activity, and above 5,725 cpm for vigorous activity. These cut points have been widely used in research studies and are useful for comparing physical activity levels across adult populations and settings. Similarly, the METs cut points from Freedson adult (1998) were adopted in this study. Participants’ percentage of time spent in different intensities of physical activity was recorded, and their total kilocalories taken over each 20-min session were calculated as energy expenditure. ActiLife software version 6.13.3 (Actigraph, United States) was used to set up the GT3X+ and to analyze these data.

#### 2.3.3. Self-efficacy and enjoyment

After each exercise session, participants responded to a 3-item self-efficacy survey and a 5-item enjoyment survey, both using a 5-point Likert-type scale (1: strongly disagree to 5: strongly agree) (Zeng et al., 2017). The self-efficacy survey items asked participants with the stem question “With regard to the [traditional aerobic dance or exergaming dance], I have confidence in…” and then rated their confidence in their ability to do well in the activity, learn skills, and perform in it. The enjoyment survey items asked participants to rate their fun doing the activity, preference to do it over other things, wanting to play it more, preferring to watch rather than play (reverse-coded) and overall likes for the activity. Item means were calculated from the answers on both surveys and used as measures of participants’ self-efficacy and enjoyment of the two types of exercise.

### 2.4. Data analysis

The research assistant entered the data electronically into SPSS while ensuring accuracy and proper documentation. Additionally, the data was kept confidential, password protected and was organized and cleaned to facilitate analysis. Furthermore, the data was stored electronically in a format that allows for exportation to statistical packages. Descriptive statistics were first calculated, followed by the dependent t-test to analyze the mean difference in energy expenditure between the two dance sessions. Subsequently, two dependent t-tests were executed to assess the difference in self-efficacy and enjoyment between the sessions. All statistical analyses were conducted in SPSS 28.0 (SPSS Inc., Chicago, IL, USA) with the significance level set at 0.05. Effect sizes (partial eta squared; η^2^) were reported for each comparison, with values of 0.01, 0.06, and 0.14 or higher interpreted as small, medium, and large, respectively (Richardson, 2011).

## 3. Results

Full demographic and baseline anthropometric information for all participants is provided in Table 1. The final sample comprised 40 young adults (Female = 33; mean age = 21.55 years, SD = 2.06). In the final sample, the majority ethnicity of participants was Han Chinese (n = 39, 97.5%) compared to the other ethnicity Dong (2.5%). In terms of college classification, 40% were sophomores, followed by graduate students (25%), seniors (22.5%), and juniors (12.5%). Additionally, only 5% of participants played an exergaming dance before participating in this study.

**Table 1:** Demographic Characteristics of Participants (*N* = 40).

Characteristics	N	% of total sample
Previous just dance experience (n some: n none)	1:39	
Gender		
Male	7	17.5
Female	33	82.5
Ethnicity		
Han	39	97.5%
Dong	1	2.5%
College Classification		
Sophomore	16	40%
Junior	5	12.5%
Senior	9	22.5%
Graduate students	10	25%
	*Mean*	*SD*
Age (years)	21.55	2.06
Height (cm)	162.68	6.71
Weight (kg)	55.61	9.77
BMI (kg/m^2^)	20.86	2.06

BMI, body mass index.

Data screening was conducted prior to the main analysis. The result yielded that all study variables were normally distributed, meaning no outliers existed in the present study. In general, the Chinese college students displayed relatively moderate to high self-efficacy and perceived enjoyment toward both dance activities, as mean scores for their self-efficacy and perceived enjoyment were above the midpoint (i.e., 3 for self-efficacy and perceived enjoyment). The participants yielded over 100 kilocalories in both dance activities in the 20 min sessions. Interestingly, these Chinese college students showed large variability in the percentage of time spent in moderate-to-vigorous physical activity, ranging from 17.50 to 69.17% for the traditional dance class, and from 10.83 to 80.00% for the exergaming dance class. They also displayed large variability in the percentage of time spent in light physical activity, ranging from 19.17 to 67.50% for the traditional dance class, and from 16.67 to 76.67% for the exergaming dance class. Additionally, the participants demonstrated large variability in the percentage of time spent in sedentary behavior, ranging from 1.60 to 41.05% for the traditional dance class, and from 3.33 to 41.09% for the exergaming dance class.

When comparing participants’ energy expenditure between exergaming aerobic dance and traditional aerobic dance (see Table 2), participants had similar kilocalories in traditional aerobic dance as they did in exergaming dance (*p* > 0.05) during the 20 min sessions. Also, no significant difference was observed for self-efficacy across both groups (*p* > 0.05). The results, however, indicate a significant main effect reported between two sessions for perceived enjoyment. In detail, traditional aerobic dance showed significantly lower enjoyment (*t* = −1.83; *p* < 0.05) compared to exergaming aerobic dance with a large effect being found.

**Table 2:** Descriptive statistics for outcome variables (*N* = 40).

	Traditional dance		Exergaming dance		*p*	η^2^
	*Mean*	*SD*	*Mean*	*SD*		
*Energy expenditure*	106.76	35.63	104.14	28.87	0.65	0.01
*Self-efficacy*	4.05	0.61	3.95	0.80	0.30	0.03
*Enjoyment*	3.61^a^	0.54	3.96	0.65	0.04	0.30

a significantly less than exergaming dance (*p* < 0.05).

SD, standard deviation.

## 4. Discussion

In this study, the energy expenditure, self-efficacy, and enjoyment of Chinese young adults were compared while they practiced exergaming dance and traditional aerobic dance. The results indicate that Chinese college students used the same energy while doing either exergaming dance or traditional aerobic dance, and the self-efficacy was similar in both activities. Notably, participants enjoyed exergaming dance significantly more than traditional aerobic dance.

In terms of energy expenditure, our data illustrated that college students’ energy expenditure in exergaming dance did not differ significantly from that of traditional dance. It is essential to investigate if this game-like exercise produces a similar physiological response to traditional aerobic dance, since increased energy expenditure during physical activity is essential for burning calories (Gao et al., 2013; Malone et al., 2019). This examination would build a body of evidence to determine if this form of exergaming can be used to promote health in young adults. Energy expenditure in exergaming is often compared to traditional aerobic exercise because it can provide many of the same benefits with some additional advantages (Hwang et al., 2019; Çakir-Atabek et al., 2020; Park et al., 2020; Ferreira et al., 2022). Exergaming typically requires the user to physically engage with the game (e.g., by hopping or jumping) just like traditional aerobic exercise does (McDonough et al., 2018; Oppici et al., 2022; Pasco and Roure, 2022; Sousa et al., 2022). This physical engagement can be seen as a form of exercise and has been shown to increase energy expenditure in some cases.

We found that young adults demonstrated similar self-efficacy in exergaming dance as they did in traditional aerobic dance. The finding fails to provide support for the second hypothesis and is inconsistent with the findings in the pediatric population (Gao et al., 2013). Adults’ self-efficacy of exergaming is similar to traditional aerobic exercise because both activities require the same physical and mental effort to be successful. The physical exertion necessary for exergaming is similar to that of traditional aerobic exercise, as both activities involve intense physical movement and require a great deal of endurance (McDonough et al., 2018; Liu et al., 2019; McDonough et al., 2020). Mental effort is also similar, as both activities require focus and concentration to reach desired results (McDonough et al., 2020; da Silva et al., 2022; Meulenberg et al., 2022). Additionally, both activities can improve physical health, as they both promote increased heart rate, improved cardiovascular health, and increased muscular strength and endurance. Ultimately, adults’ self-efficacy of exergaming dance and traditional aerobic exercise are similar because they both require physical and mental effort to be successful.

The finding is in line with our hypothesis, as exergaming might be more attractive and enjoyable than traditional aerobic exercise. It is congruent with previous research literature comparing aerobic exercise modalities in pediatric and adult populations (Gao, 2012; Gao et al., 2013, 2014). Adults often find exergaming more enjoyable than traditional aerobic exercise because it adds an element of fun and competition (McDonough et al., 2018; Morais et al., 2022). Exergaming combines the physical activity of traditional aerobic exercise with the engagement and interactivity of video games, making it an enjoyable and immersive experience. Exergaming also encourages exploration and encourages players to try new activities, as there is a wide variety of exergames to choose from. Because of the fun and engagement of exergaming, adults are more likely to commit to their fitness goals and enjoy the process of working out (McDonough et al., 2018; Seo et al., 2023). In addition, exergaming often includes features that may increase motivation to play, such as attractive visuals or rewards for reaching certain goals. This can make exergaming more enjoyable than traditional aerobic exercise and can lead to longer, more consistent exercise sessions (McDonough et al., 2018; Liu et al., 2019). Overall, exergaming can offer similar benefits to traditional aerobic exercise while providing additional features to increase motivation and enjoyment.

Overall, exergaming dance has become an increasingly popular form of exercise for young adults. This type of exercise combines physical activity with interactive gaming elements to create a fun and engaging workout experience. Compared to traditional aerobic exercise, exergaming dance offers similar energy expenditure and self-efficacy, but higher enjoyment and increased motivation for physical activity. Additionally, exergaming dance is a novel way to exercise, providing a refreshing break from the monotony of ordinary workouts. Ultimately, exergaming dance is a great way for young adults to get active, stay fit, and have fun. This study is unique in that it measures the effects of two different types of dances (exergaming dance and traditional aerobic dance) on energy expenditure, self-efficacy, and enjoyment in Chinese college students. However, several limitations should be noted. These include not examining differences between male and female students, unbalanced sample size between male and female participants, not collecting information on physical activity and diet history, and the fact that this is only a one group pre-test and post-test experiment, meaning cause and effect relationships cannot be established. The dominance of one gender in the study might affect generalization. In addition, we did not conduct the power calculation to determine the sample size in this preliminary study, and thus the sample size may have compromised the results. Also, although we screened and sorted the data, we did not perform the absence of outliers testing and there is a chance for us to omit a few outliers in this study. To gain a better understanding of the effects of exergaming dance, further research using true experimental designs and decent sample is needed.

## 5. Conclusion

This study suggests that exergaming aerobic dance can have a beneficial effect on the enjoyment of Chinese young adults. Specifically, energy expenditure in exergaming is comparable to traditional aerobic exercise in Chinese young adults. Exergaming is an effective form of exercise because it encourages the user to exert energy and physical effort to achieve a desired outcome. In addition to burning calories, exergaming can be an enjoyable and motivating form of exercise for those who prefer to stay active without the monotony of traditional aerobic exercise. Exergaming can also provide a more immersive experience than traditional aerobic exercise, as users can interact with the game, which can lead to increased engagement and motivation while exercising (McDonough et al., 2018; Liu et al., 2019). The results suggest that young adults could be more inclined to take part in gaming-based exercise if they expend the same amount of energy as they would in traditional aerobic exercise. Further research is needed to determine which type of exergaming is most beneficial to young adults’ health and well-being when engaging in physical activities.

## Data availability statement

The raw data supporting the conclusions of this article will be made available by the authors, without undue reservation.

## Ethics statement

The studies involving humans were approved by Ethics review committees at Hunan Normal University. The studies were conducted in accordance with the local legislation and institutional requirements. The participants provided their written informed consent to participate in this study.

## Author contributions

The author would like to thank the co-authors for their help to complete this study. During the construction of this study, DG and CG played a role in data sorting and writing the article. WL and XL played a role in data collection and paper editing. JO and SR played a role in helping write the article. ZG played a role in developing the research ideas, overseeing data collection and analysis, and revising the article. All authors contributed to the article and approved the submitted version.

## Conflict of interest

The authors declare that the research was conducted in the absence of any commercial or financial relationships that could be construed as a potential conflict of interest.

## Publisher’s note

All claims expressed in this article are solely those of the authors and do not necessarily represent those of their affiliated organizations, or those of the publisher, the editors and the reviewers. Any product that may be evaluated in this article, or claim that may be made by its manufacturer, is not guaranteed or endorsed by the publisher.
